# LightGBM Indoor Positioning Method Based on Merged Wi-Fi and Image Fingerprints

**DOI:** 10.3390/s21113662

**Published:** 2021-05-25

**Authors:** Huiqing Zhang, Yueqing Li

**Affiliations:** 1 Faculty of Information Technology, Beijing University of Technology, Beijing 100124, China; zhq@bjut.edu.cn; 2 Engineering Research Center of Digital Community, Ministry of Education, Beijing 100124, China; 3 Beijing Laboratory for Urban Mass Transit, Beijing 100124, China

**Keywords:** Wi-Fi fingerprint, LBP features, ensemble-learning, merged fingerprint

## Abstract

Smartphones are increasingly becoming an efficient platform for solving indoor positioning problems. Fingerprint-based positioning methods are popular because of the wide deployment of wireless local area networks in indoor environments and the lack of model propagation paths. However, Wi-Fi fingerprint information is singular, and its positioning accuracy is typically 2–10 m; thus, it struggles to meet the requirements of high-precision indoor positioning. Therefore, this paper proposes a positioning algorithm that combines Wi-Fi fingerprints and visual information to generate fingerprints. The algorithm involves two steps: merged-fingerprint generation and fingerprint positioning. In the merged-fingerprint generation stage, the kernel principal component analysis feature of the Wi-Fi fingerprint and the local binary pattern features of the scene image are fused. In the fingerprint positioning stage, a light gradient boosting machine (LightGBM) is trained with mutually exclusive feature bundling and histogram optimization to obtain an accurate positioning model. The method is tested in an actual environment. The experimental results show that the positioning accuracy of the LightGBM method is 90% within a range of 1.53 m. Compared with the single-fingerprint positioning method, the accuracy is improved by more than 20%, and the performance is improved by more than 15% compared with other methods. The average locating error is 0.78 m.

## 1. Introduction

Location-based services deliver excellent research and commercial value and have become a common object of research. The Global Navigation Satellite System (GNSS) provides reliable location services outdoors. With the expansion of urban areas, human activities in indoor environments are becoming increasingly abundant, and the demand for indoor positioning services is increasing. Meanwhile, smartphones that integrate wireless, visual, and accelerometer sensors can facilitate indoor positioning services [1]. However, owing to the complex and variable natures of indoor environments, the large-scale application of indoor positioning solutions has yet to be achieved. Researchers have used a variety of indoor signals for positioning, including wireless local area network (WLAN) facilities widely distributed in indoor environments, cellular networks [2], Bluetooth [3], radio-frequency identification [4] and other radio frequency signals, microelectromechanical system gyroscopes [5], ultra-wideband (UWB) [6,7], laser ranging [8], and visual information [9]. Wi-Fi fingerprint positioning does not require the distances and angles to be known in advance; however, it is seriously affected by indoor multipath effects. Cellular networks are mainly used for smartphone positioning; however, their accuracy is generally low because of problems such as time synchronization; with the development of 5G communication network technology, this method is expected to achieve higher accuracies. Although image-based positioning technology offers high accuracy, it suffers from occlusion, illumination, and blur. Bluetooth positioning offers the advantages of low power consumption, close range, and wide applicability, though its stability is poor. The UWB signal has strong penetrability and high security, and it can integrate positioning and communication functions; however, owing to its high cost, it has not yet been widely implemented. Individual positioning methods struggle to meet the high-precision pedestrian self-positioning requirements, despite their portability, cost, and environmental adaptability [10]. The fusion positioning method exploits the complementary capacities of different sensors to obtain information and provide a richer description of indoor locations; thus, it has become a research hotspot in recent years. For example, the channel-state-information/magnetic-field-strength fusion positioning method proposed by Li et al. [11] and Simon et al. [12] uses a visual inertial positioning algorithm to automatically collect Bluetooth signal strength data and reduce the labor density of the earlier data-collection methods based on wireless signal positioning. Single-signal-source positioning technologies rely on the stability of this type of signal, which is extremely difficult to maintain in a complex indoor environment. The motivation for fusing visual and Wi-Fi signals for positioning applications is to improve the adaptability of the positioning system to indoor environments. This can prevent positioning failures caused by a single signal fluctuation. The present work proposes a new smartphone-based indoor positioning method that uses the scene image and received signal strength (RSS) value of the WLAN access point (AP) as the input of the merge positioning system to realize high-precision pedestrian self-positioning. This research can also be used for the auxiliary positioning of indoor service robots and in business advertising campaigns.

Scene images and Wi-Fi fingerprint positioning approaches must consider two key issues: (1) the extraction of key positioning information from the Wi-Fi fingerprints and scene images, and (2) the dimensional unification of image information and Wi-Fi fingerprints. The focus of this article is to propose a new combined fingerprint that describes location information using visual and Wi-Fi signals to realize combined-fingerprint positioning. The achieved positioning accuracy is more than 20% higher than that obtained using Wi-Fi or visual positioning methods alone; meanwhile, it achieves a faster running speed. It takes less than 2 s to obtain the predicted position coordinates from the feature extraction results; thus, this method can meet the demands of real-time positioning. The main contributions of this study are as follows:

- We propose a merged location fingerprint based on Wi-Fi fingerprints and scene image features. Of these, Wi-Fi fingerprint features are obtained by extracting effective positioning information from the original Wi-Fi fingerprint using the kernel principal component analysis (KPCA) method; next, scene image features are extracted by local binary patterns (LBPs). The image data are transformed into structured data so that the scene information and Wi-Fi fingerprint can jointly describe the spatial location in the same dimension, which reduces the storage space occupied by the merge fingerprint library.
- Based on the merged location fingerprint, a light gradient boosting machine (LightGBM), which can effectively process structured data, is used to construct an indoor positioning model. This positioning model can quickly and accurately obtain positioning results, and is easy to implement on smartphone platforms with limited computing resources. Our experiments prove that the proposed method is simple and effective.

The structure of this paper is as follows: Section 2 introduces the relevant research regarding the use of scene images and Wi-Fi fingerprints in indoor positioning; Section 3 introduces the merged fingerprint generation procedure and the construction of indoor positioning models to realize individual positioning; Section 4 presents the experimental results and analysis; finally, Section 5 summarizes the research and considers future research directions.

## 2. Related Work

Indoor positioning technology provides users with positioning functions in indoor public places. The main challenges to this technology are signal fluctuations caused by complex and diverse indoor environments, the construction and updating of accurate maps, and the integration of different technologies and signal sources. In recent years, indoor positioning systems that rely on the multiple sensors and computing resources offered by smartphones have received widespread attention [13,14,15]. Smartphone-based positioning systems have the advantages of being cheap and portable, making them suitable for pedestrian self-positioning. At present, smartphone-based indoor positioning methods are primarily divided into two categories [16]: positioning by means of facilities deployed in the environment (e.g., Wi-Fi and Bluetooth) and self-positioning systems without infrastructure (e.g., pedestrian dead reckoning (PDR)). Basem et al. [17] proposed an indoor navigation system for blind users; within a fuzzy logic framework, the Euclidean distance was calculated using the received signal strength value of the Bluetooth low-energy beacon and the set distance from the current beacon to the fingerprint point. They achieved an average positioning error of only 0.43 m; however, larger or more complex indoor environments may require more beacons. Zeng et al. [18] integrated optical sensor, magnetic sensor, and GNSS signals into a navigation algorithm to achieve seamless positioning continuity and accuracy between the two environments. ViNav, proposed by Dong et al. [19], is a scalable and cost-effective system; it uses automatic structure technology to reconstruct a three-dimensional model of the indoor environment from crowdsourced images and locate points of interest within the three-dimensional model; it can achieve user positioning with an error of less than 1 m in under 2 s. Lu et al. [20] proposed an inertial navigation system/PDR integrated navigation method based on motion recognition; this calculates pseudo-heading measurements from motion recognition results, thereby effectively suppressing the heading angle drift; however, this method cannot provide long-term high accuracy, owing to error accumulation.

### 2.1. Indoor Positioning Technology Based on Wi-Fi Signals and Visual Information

Because Wi-Fi signals are readily available in public indoor environments, Wi-Fi-based indoor positioning methods are the most popular [21]. Currently, the commonly used Wi-Fi positioning models are the trilateral method [22] and fingerprint method [23]. The Wi-Fi fingerprint is composed of the Wi-Fi received signal strength indicator (RSSI) of different APs for known location reference points (RPs). Guo et al. [24] constructed a group of merged fingerprints consisting of RSS, signal-strength-difference, and hyperbolic position fingerprints, fully exploiting the complementarity of fingerprints. They simultaneously proposed an optimal classifier selection algorithm to realize precise positioning and in-depth mining of location information in Wi-Fi fingerprints. The positioning framework INTRI, proposed by He et al. [25], introduces the idea of trilateral positioning using fingerprint recognition and estimates the user’s position from the RSSI contours of three APs. Although wireless signal positioning technology can facilitate self-contained systems, it is still very difficult to accurately model the multipath effects and personnel-induced fluctuations caused by complex environments.

Following improvements in the image-processing performances of mobile smart devices, vision-based indoor positioning methods have also received widespread attention. Visual positioning systems can be divided into two categories. The first category analyzes and processes the sequence images input using mobile visual sensors and estimates the position and pose of the carrier. One representative algorithm is visual odometry, which is primarily used as a front-end application for simultaneous localization and mapping. Another type of visual-sensor-based positioning algorithm uses a fixed-position vision sensor to determine the position of the target to be measured in the image. This is typically implemented using target tracking and detection algorithms. One representative application is the Easy Living System of the Microsoft Research Institute [26]. Vedadi et al. [27] proposed a system for automatically generating an image-positioning database based on an automatic Wi-Fi fingerprint acquisition system. This method uses a known map of the collection area, supplemented by movement information collection. The system receives the data recorded by the mobile device. The frame sequence of the unmarked position automatically uses the map coordinates to mark the video frame according to the motion information. Walch et al. [28] used GoogleNet to extract image features and long short-term memory to estimate the position of the camera in combination with time-series information, and achieved better positioning results in the case of less or no texture. However, approaches that use only visual information for positioning are limited by the amount of texture information, occlusion, and moving speed. The real-time incoming sequence images and deep network used for processing images also place higher requirements on the performance of smartphones.

### 2.2. Indoor Positioning Method Based on Wi-Fi and Image Merging

The aim of fusing Wi-Fi and vision-based indoor positioning methods is to achieve complementary advantages, enrich the descriptiveness of location information, and improve positioning accuracy. The RAVEL system proposed by Papaioannou et al. [29] used wireless signals to improve the accuracy of the visual monitoring system, for tracking and positioning people. Jiao et al. [30] proposed an optimized edge particle filter algorithm to fuse time & code division-orthogonal frequency division multiplexing and image feature positioning information. Antonio et al. [31] used the Wi-Fi signal strength, digital compass, and accelerometer information measured by the smartphone to delineate a rough position; then, they matched the captured image with the three-dimensional model of the sub-region, reducing the number of smart terminals. Inspired by RGB-D cameras, Alexandre et al. [32] used Wi-Fi information to expand RGB data to track and locate people; that is, they used the RGB information to estimate the center coordinates of the camera and Wi-Fi information to estimate the depth. Jiao et al. [33] used deeply fused wireless signals and images for positioning; this method converts the wireless signals received within a certain period of time into frequency-domain signals via a wavelet transform; then, it generates W-images and uses a scale-invariant feature transform to compare them. The image performs feature extraction, merges with the LBP features extracted from the smartphone camera image, forms a dictionary, and uses the lasso method to match and locate. Hu et al. [34] proposed a new Wi-Fi and visual integrated fingerprint, referred to as Wi-Vi fingerprint, which was used for accurate indoor positioning. The method uses the exit signs in a building to calculate the image fingerprints and performs rough positioning via Wi-Fi fingerprint matching, image matching positioning, and refined positioning to obtain the final position estimation. Milan et al. [35] discussed a merge strategy based on WLAN and images, using the extended naive Bayes method and a speeded-up robust features algorithm based on a hierarchical vocabulary tree to localize the WLAN and image, respectively; then, they proposed a particle filter position estimation method from the two perspectives of features and localization results. The filtering position estimation method had an improved adaptability to different scenes. The cost of image processing and storage was much higher than that of Wi-Fi fingerprints, and step-by-step positioning increased the operating time of the system. Jiao et al. [36] proposed an intelligent deep learning fusion architecture to construct an RGB-WM image that combines visual, Wi-Fi, and inertial information before extracting invariant features using an improved convolutional neural network. Offline positioning was achieved by transplanting trained weights to the mobile devices. The positioning accuracy of this algorithm was less than 1.23 m. This method provides an excellent framework, though the construction and feature extraction of fusion images places higher requirements on the computing power of mobile devices. Realizing real-time positioning represents a significant challenge.

To summarize, positioning schemes requiring additional experimental facilities and equipment are less convenient and economical than those without such infrastructure. In contrast to other visual and Wi-Fi information fusion methods, this study processes a huge image into a vector instead of transforming the Wi-Fi signal into a complex image. Such processing makes the positioning feature more concise and effective. Compared with the deep learning model, LightGBM is lightweight and more interpretable; it provides a new solution for real-time positioning on mobile platforms with limited computing power. This research is based on smartphones, using existing wireless APs and scene images in a public indoor environment to achieve positioning. The innovations of this work are as follows: (1) It unifies Wi-Fi and images into the same data dimension, (2) it uses merged location fingerprints to describe scene location information, and (3) it uses the LightGBM algorithm to perform regression mapping between merge fingerprints and spatial location coordinates.

## 3. Merge Fingerprint LightGBM Indoor Positioning Algorithm

**Figure 1:**
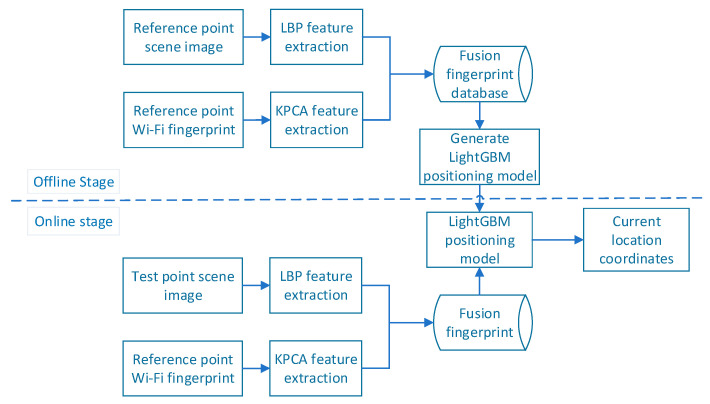
Flow chart of merged location fingerprint positioning system.

The merged fingerprint proposed in this study includes Wi-Fi fingerprint features and scene image features. Figure 1 presents a flow chart of the merged-fingerprint positioning system. The Wi-Fi fingerprint feature is obtained using the KPCA method, and the scene image feature is represented by the LBP feature histogram (i.e., the LBPH). Both Wi-Fi signals and scene images have their own uniqueness; that is, the merged fingerprint and location information correspond uniquely. The flow chart of the merged location fingerprint positioning system is shown in Figure 1; it is divided into offline and online stages.

First, in the offline stage, the RPs are divided into the experimental area $P=\{P_{1},P_{2},\ldots ,P_{n}\}$, where *P* denotes the set of RPs, and *n* is the total number of sampling points. The location feature is extracted from the scene image and Wi-Fi RSSI obtained at the RP, the merged fingerprint database is generated, and the LightGBM positioning model is trained. In the online stage, the same processing is performed on the data collected at the test point to generate a merged fingerprint of the point to be located, and the trained positioning model is used to predict the current position coordinates.

### 3.1. Extract the KPCA Features of Wi-Fi Fingerprints

The Wi-Fi fingerprint is collected by the mobile device, and it includes the media access control address of the access point and the corresponding RSSI. The dimension of the Wi-Fi fingerprint depends on the number of access points that can be received in the localization area; hence, the Wi-Fi fingerprint is a type of high-dimensional data. At the same time, Wi-Fi fingerprints are closely related to location, albeit non-linearly. Wi-Fi fingerprints are time-varying, high-dimensional nonlinear data. Directly using the original Wi-Fi fingerprint to identify the location is inefficient, and noise interference arises. In this research, KPCA was selected for Wi-Fi fingerprint characteristics, to reduce fingerprint dimensions and extract key positioning features.

KPCA represents a non-linear extension of PCA; it can realize the nonlinear dimensionality reduction of, and feature extraction from, data. The basic idea is to use the kernel function to map a linearly inseparable dataspace to a high-dimensional space, making it linearly separable in the high-dimensional space before performing PCA.

The Wi-Fi fingerprint sample set is $Z{=\{(R}_{iq},P_{i}{)}_{q=1}^{Q}{\}}_{i=1}^{n}$, q=1,2,…,Q. $R_{iq}$ denotes the *q*-th fingerprint at the *i*-th position, *Q* is the number of fingerprint samples at each RP, *n* is the number of RPs, and *N* is the total number of fingerprint samples, N=n×Q. $R_{iq}=[RSSI_{i1},RSSI_{i2},\ldots \ldots ,RSSI_{ik}]$, $RSSI_{ij}$ (j=1,…,K) indicates that the RSSI value from the j-th AP is received at the position of RP *i*. $P_{i}=(x_{i},y_{i})$ denotes the physical position coordinate of the fingerprint point *i*. Let $W=(R_{11}^{\prime },R_{12}^{\prime },\ldots \ldots ,R_{nQ}^{\prime })$, where *W* is the standardized Wi-Fi fingerprint data. When mapping *W* to a high-dimensional space via the mapping function Φ, the mapping function is unknown. In the dataset *W*, each sample $R_{iq}^{\prime }$ is a K-dimensional column vector, and there are N samples in *W*. The space containing the *K* × *N* matrix *W* is referred to as the input space. The KPCA feature extraction process for WiFi fingerprints is as follows:

We use a nonlinear mapping Φ to map the vector w in *W* to the D-dimensional feature space F, as follows:

(1) $$\Phi (W):R_{iq}^{\prime K}\to R_{iq}^{\prime D},D\gg K.$$

After mapping, a new *D* × *N* matrix Φ(*W*) in the feature space is obtained, as

(2) $$\Phi (W)=[\Phi (R_{11}^{\prime }),\Phi (R_{12}^{\prime }),\ldots \ldots ,\Phi (R_{nQ}^{\prime })],$$

where $\Phi (R_{iq}^{\prime })$ represents the mapping of $R_{iq}^{\prime }$ in the high-dimensional feature space. We perform PCA analysis on Φ(W) to obtain the KPCA features of the Wi-Fi fingerprint $w=[w_{11},w_{12},\ldots \ldots ,w_{nQ}]$.

Through the KPCA processing, the original position fingerprint space of the *K × N* order can be transformed into a *K × m* feature-position fingerprint space. $Z^{\prime }{=\{(w}_{iq},P_{i}{)}_{q=1}^{Q}{\}}_{i=1}^{n}$ is the Wi-Fi fingerprint KPCA feature dataset, where the dimension is *m*. The fingerprint feature dimension *m* has a larger influence on the model prediction accuracy. Therefore, the fingerprint feature dimension must be selected in the offline training stage to achieve the optimal positioning effect.

### 3.2. Extract Image LBP Features as Image Fingerprints

The location fingerprint can be any feature that facilitates location discrimination, and it can have diverse types. In this study, the LBPH is used as the image fingerprint to describe the location information together with the Wi-Fi fingerprint. Positioning methods based on visual information are often affected by illumination, occlusion, and shooting angles. LBP is a highly discriminative texture operator with significant advantages in terms of gray-level and rotation invariance. It is widely used in target detection to describe image texture features. Ojala et al. [37] proposed a uniform pattern to reduce the dimensionality of the LBP operator model. This is defined such that when the cyclic binary number corresponding to a certain LBP has at most two transitions (from 0 to 1 or from 1 to 0), the binary corresponding to the LBP is a uniform pattern class. This study uses the uniform-pattern-based rotation-invariant LBP feature to describe the scene contour and texture information, and it calculates it according to

(3) $$U(LBP_{l,r}^{u})=\left|s(g_{l-1}-g_{c})-s(g_{0}-g_{c})\right|+\sum_{l=1}^{L}\left|s(g_{l}-g_{c})-s(g_{l-1}-g_{c})\right|,$$

(4) $$LBP_{l,r}^{2}=\left\{\begin{array}{c} \sum_{l=0}^{L-1}s(g_{l}-g_{c}),\overset{}{}\overset{}{}if\;U(LBP_{l,r}^{u})\leq 2\\ L+1,{}_{}otherwise \end{array}\right.,$$

(5) $$s(g)=\left\{\begin{array}{c} 1,if\;g\geq 0\\ 0,if\;g\leq 0 \end{array}\right.,$$

where *L* represents the number of pixels in the neighborhood, $g_{c}$ is the gray value of the central pixel, and $g_{l}$ is the gray value of the neighborhood pixel. $s(g_{l}-g_{c})$ compares the sizes of $g_{l}$ and $g_{c}$; if $g_{l}$ exceeds $g_{c}$, the value of $s(g_{l}-g_{c})$ is 1; otherwise, it is 0. $U(LBP_{l,r}^{u})$ is the metric of the rotation-invariant uniform pattern, which represents the number of transitions from 0 to 1 or from 1 to 0. $LBP_{l,r}^{2}$ calculates the unique label corresponding to the type of LBP rotation-invariant equivalent mode operator whose number of transitions (from 0 to 1 or from 1 to 0) does not exceed 2. s(g) is a symbolic function. This study uses $LBP_{8,1}^{2}$ to calculate the type of rotation-invariant uniform pattern. We perform histogram statistics on the number of pixels in the LBP rotation-invariant uniform pattern operator category of the entire image, to obtain a ten-dimensional LBP feature histogram vector. The uniform-pattern-based rotation-invariant LBP operator guarantees the stability of the image fingerprint. At the same time, the LBP image fingerprint is a type of structured data that can be combined with a Wi-Fi fingerprint to form a merged fingerprint.

### 3.3. Build a Merged Fingerprint

**Figure 2:**
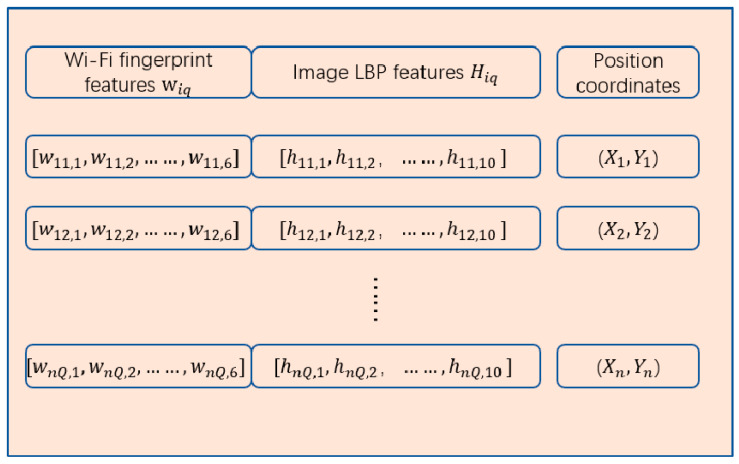
Merged fingerprint database.

The first fusion of the image and Wi-Fi fingerprints involves direct stitching. The merged fingerprint is obtained by merging the KPCA features of the Wi-Fi fingerprint and image fingerprint after dimensionality reduction. Generally, Wi-Fi fingerprints are taken at the same location through multiple collections of the mean or median values. The merged fingerprint proposed in this study uses multiple scene images from the same RP position. Therefore, in the experiment, the same number of Wi-Fi fingerprint data as the scene image were also collected at the same RP position, and the corresponding scene image features and Wi-Fi fingerprint features were spliced together. The merge fingerprint database is shown in Figure 2; it contains the KPCA features of the Wi-Fi fingerprints, a total of six dimensions, and the scene image LBP features with a length of 10 dimensions. The form of the merge fingerprint is $d_{iq}={\left\{{\left(w_{iq}^{*},H_{iq}\right)}_{q=1}^{Q}\right\}}_{i=1}^{n}$, and the merge fingerprint data set is $D={\left\{{\left(d_{iq},P_{i}\right)}_{q=1}^{Q}\right\}}_{i=1}^{n}$, where $w_{iq}^{*}$ is the Wi-Fi fingerprint feature of the location point *i*, $H_{iq}$ is the *q*-th image fingerprint at position *i*, and $P_{i}$ is the coordinate of the location point *i*. The merged fingerprint database is shown in Figure 2.

### 3.4. Establish LightGBM Positioning Model

LightGBM is an ensemble learning framework based on gradient boosting decision trees that was developed by Microsoft Research. It uses decision trees as the base learner to continuously fit the residuals of the current learner, and it iteratively trains the model using a forward distribution algorithm. Each iteration seeks to minimize the loss function. LightGBM uses a histogram-based segmentation algorithm to replace the pre-sort traversal algorithm, and it reduces the number of samples and features through gradient-based, one-side sampling and exclusive feature bundling (EFB). This model offers the advantages of fast and high performance. 

The second merge of the merge fingerprints is performed using the EFB algorithm in LightGBM. ‘Exclusive feature’ refers to the fact that some features rarely have non-zero values simultaneously, and these features are bundled together to form a new feature, which is used to reduce the number of features and improve training speeds. The EFB algorithm applies the idea of graph building, uses features as nodes, connects edges between non-mutually exclusive features, and then identifies all bundled feature sets from the graph. This problem is an NP-hard problem, and EFB uses a greedy strategy to solve it. This allows a small number of samples between features that are not mutually exclusive and sets a maximum conflict threshold K. The time complexity of the EFB algorithm is O(n^2^).

The process of establishing the fusion fingerprint LightGBM positioning model is as follows:

First, the merged fingerprint dataset *D* is used as the input, and the first boosted tree $f_{0}(d_{iq})$ is initialized as

(6) $$f_{0}(d_{iq})=\arg\min_{c}\sum_{i=1}^{n}\sum_{q=1}^{Q}L(P_{i},c),$$

where $P_{i}$ represents the spatial position coordinates of the *i*-th collection point; c is the output value of the leaf node of the promotion tree, which is the value that minimizes the loss function (i.e., the predicted value of the position coordinates of the *i*-th collection point); and *L* is the loss function.

Suppose the decision tree obtained from the *t*−1 iteration is $f_{t-1}(d)$, and the loss function is $L(P_{i},f_{t-1}(d))$. Then, the purpose of the *t*-th iteration is to identify the base learner $T(d,\theta_{t})$ and minimize the loss function $L(P_{i},f_{t}(d))=L(y,f_{t-1}(d)+T(d,\theta_{t}))$.

This article uses the mean square error loss function, which is

(7) $$L(y,f_{t-1}(d)+T(d,\theta_{t}))=\frac{1}{2}{\left[y-f_{t-1}(d)-T(d,\theta_{t})\right]}^{2}={\left[\tau -T(d,\theta_{t})\right]}^{2},$$

where $\tau =P_{i}-f_{t-1}(d)$ is the residual. The decision tree fits the residual of the current learner at each iteration. Typically, the value of the negative gradient of the loss function in the current learner is used as an approximate value:

(8) $$\tau_{iq,t}\approx -{\left[\frac{\partial L(P_{i},f(d))}{\partial f(d)}\right]}_{f(d)=f_{t-1}(d)}.$$

The residual is taken as the new true value of the sample, and we use ${\left\{{\left(d_{iq},\tau_{iq,t}\right)}_{q=1}^{Q}\right\}}_{i=1}^{N}$ as the training data to obtain the decision tree $f_{t}(d)$, where the set of leaf nodes is $C_{tj}$, j=1,2,……,J. For each leaf node, we calculate the best-fit value $c_{tj}$ as follows:

(9) $$c_{tj}\approx \arg\min_{c}\sum_{d_{iq}\in C_{tj}}L(P_{i},f_{t-1}(d)+cf_{t}(d)).$$

We update positioning model $f_{t}(d)$, as follows:

(10) $$f_{t}(d)=f_{t-1}(d)+\sum_{j=1}^{J}c_{tj}I(d\in C_{tj}).$$

We obtain the final positioning model as:

(11) $$f_{T}(d)=f_{0}(d)+\sum_{t=1}^{T}\sum_{j=1}^{J}c_{tj}I(d\in C_{tj}).$$

**Figure 3:**
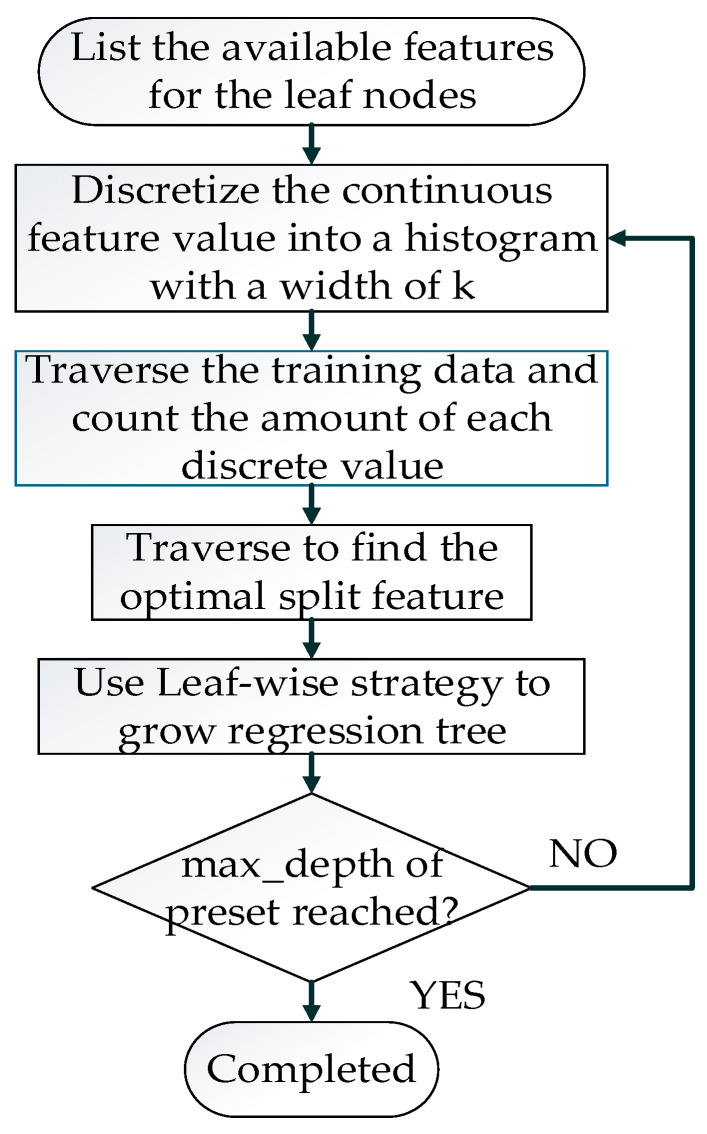
Process of training a regression tree in LightGBM.

The process of training a regression tree in LightGBM is shown in Figure 3.

The pseudocode of the algorithm that identifies the optimal split point mentioned in Figure 3 is shown in Algorithm 1. The time complexity of the histogram algorithm for calculating the split gain is O(bin*features), where “bin” denotes the number of bins for each feature. Compared with other decision tree algorithms that use pre-sorting algorithms, the time complexity of the pre-sorting algorithm is greatly reduced, being expressed as O(data*features). The bin is much smaller than the data.

Algorithm 1 Identifying the optimal splitting point algorithm of the histogram.
**Algorithm 1** BestSplitByHistogram Algorithm1**Input:** *d*: training data, max_depth2**Input:***m*: merger fingerprint dimension3*nodeSet* = {0} #tree nodes in current level4*rowSet* = {{0,1,2,...}} #data indices in tree nodes5**for** *i* = 1 **to** max_depth6
**for** *node* in *nodeSet* **do**7

usedRows = *rowSet*[node]8

**for** *j* = 1 **to** m **do**9


*H* = new Histogram()10


#Build histogram11


**for** *k* in *usedRows* **do**12



bin = d.s[j][k].bin13



*H*[bin].g += d.g[j] #Sum of gradients in each bin14



*H*[bin].n += 1 #Sum of samples in each bin15


Find the best split on histogram H.16
Update rowSet and nodeSet according to the best split points

In this study, we constructed the LightGBM positioning model for merged fingerprint dataset D according to the X and Y coordinates. The establishment of the fusion fingerprint LightGBM model proceeds as shown in Algorithm 2.

Algorithm 2 Establishing the merged fingerprint LightGBM model.
**Algorithm 2** LightGBM localization algorithm based on merged fingerprint1**Input:** imgSet, wifiFingerprintSet, Rpnum2wifi_KPCA = [[]]3imgFingerprint = [[]]4mergeFP = [[]]5wifi_KPCA = KPCA(wifiFingerprintSet)6**for** *n* = 1 **to** Rpnum 7
for *q* to n do8

imgFingerprint[*n*][*q*] = LBP(imgSet[*n*][*q*])9

mergeFP[*n*][*q*] = [wifi_KPCA[*n*][*q*], imgFingerprint[*n*][*q*]]10XpreModel = LightGBM.train(mergeFP,Xcoordinates)11YpreModel = LightGBM.train(mergeFP,Ycoordinates)

## 4. Experimental Results and Analysis

### 4.1. Experimental Setup

**Figure 4:**
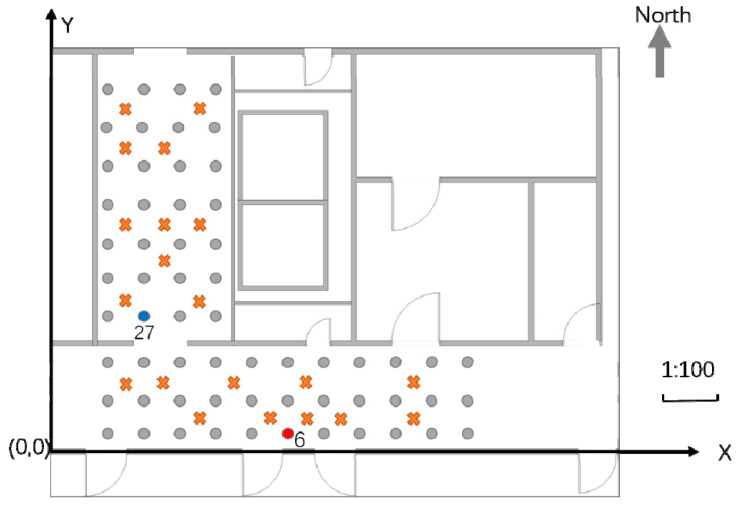
Plan view of the experimental area.

This study verifies the positioning performance of the proposed algorithm in a teaching facility environment. The experiment was conducted in the corridor and elevator room on the tenth floor of the Science Building of Beijing University of Technology. The area of the experimental environment was 10 m × 7 m. A partial plan view is presented in Figure 4. In the figure, the distance between two adjacent points in the X direction is 0.85 m, and the distance in the Y direction is 0.7 m; the 60 dots represent RPs, and the 20 cross-shaped dots represent test points. The experiment used self-developed RSSI signal acquisition software to collect 69 APs deployed in the teaching area. A total of 20 merged fingerprints at each RP were collected in the southern, eastern, northern, and western directions. The Wi-Fi fingerprint and image acquisition device was a Mi 10 Ultra smartphone, for which the parameters are listed in Table 1. The shooting height of the scene image was ~1.5 m (the experimenter’s height was 1.7 m).

### 4.2. Data Preprocessing

**Figure 5:**
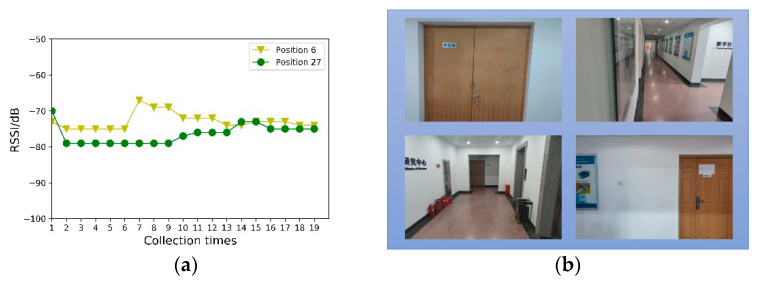
Comparison of Wi-Fi fingerprints and scene images in different locations: (**a**) The RSS value for the same AP, received at Positions 6 and 27. (**b**) The upper two pictures are the scene images at Position 6, and the lower two pictures are the scene images at Position 27.

Figure 5a denotes the RSS value of the same AP received at Positions 6 and 27. It can be observed that the Wi-Fi signal exhibits severe volatility. However, the same RSS value may appear in different positions, which causes difficulties in position discrimination. Position 6 is represented by a red dot in Figure 4, and Position 27 is represented by a blue dot. Figure 5b shows the scene images in Positions 6 and 27 at the top and bottom, respectively. Though the Wi-Fi signal strengths may appear identical, the difference between the scene images is very large, providing a multi-angle description of the position information.

The Wi-Fi fingerprint was preprocessed, the signal strengths of the AP not collected at the collection point was set to −100, and the Wi-Fi fingerprint data after KPCA dimension reduction were normalized using

(12) $$w_{iq}^{*}=\frac{w_{iq}-w_{iq,\min}}{w_{iq,\max}-w_{iq,\min}},$$

where $w_{iq,\min}$ refers to the minimum value in the sample data, $w_{iq,\max}$ refers to the maximum value in the sample data, and $w_{iq}^{*}$ is the normalized Wi-Fi fingerprint data.

We normalize the LBP feature histogram, as follows:

(13) $$h_{iq,k}=\frac{b_{k}}{\sum_{k=0}^{K+1}b_{k}}.$$

Here, $h_{iq,k}$ is the normalized result of the *K*-th type of rotation-invariant uniform pattern LBP operator, and $b_{k}$ denotes the number of pixels belonging to the *k*-th rotation-invariant uniform pattern operator class; there are ten types in total; hence, $H_{iq}=[h_{iq,1},h_{iq,2},\ldots ,h_{iq,10}]$. *b* is the normalized LBP feature vector of the *i*-th scene image.

In this study, the calculation formulas of the positioning error $e_{i}$ and average positioning error *ave* are as follows:

(14) $$e_{i}=\sqrt{{\left(x_{pre}-x_{i}\right)}^{2}+{\left(y_{pre}-y_{i}\right)}^{2}},$$

(15) $$ave=\frac{1}{M}\sum_{i=1}^{M}e_{i}.$$

$\left(x_{pre},y_{pre}\right)$ are the predicted coordinates of the positioning algorithm, $\left(x_{i},y_{i}\right)$ are the real coordinates of the test point, and *M* is the total number of samples in the test set.

Accuracy was also used to evaluate the positioning results in this study [38]; it corresponds to the error distribution of the distance between the predicted and true positions. The cumulative distribution function is typically used to measure accuracy. For an indoor positioning algorithm with identical accuracy, the faster the cumulative distribution function curve reaches the peak, the better the method performance. In practice, a percentage is generally used to calculate accuracy. For example, if the accuracy of a positioning method within 1.5 m is 90%, the cumulative distribution function of the positioning error is less than 90% within 1.5 m.

### 4.3. The Influence of KPCA Location Feature Extraction on Location Accuracy

**Figure 6:**
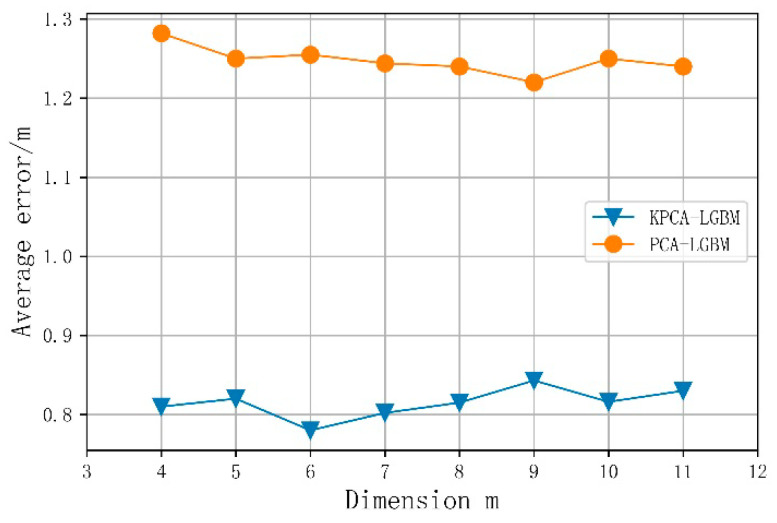
Influence of fingerprint dimension on average positioning error.

Through a selection experiment, this study verifies the ability of KPCA to reduce the noise interference of the original Wi-Fi fingerprint whilst retaining the fingerprint dimension; as shown in Figure 6, we compare the obtained results with those of the PCA algorithm, to verify the effectiveness of KPCA for processing the sparse data of Wi-Fi fingerprints. The PCA algorithm’s mapping from high- to low-dimensional spaces is linear; thus, it is difficult to effectively process Wi-Fi fingerprint information, and the positioning results of the KPCA-LGBM algorithm are significantly better than those of the PCA-LGBM one. To summarize, when the Wi-Fi fingerprint dimension *k* increases, the positioning error first decreases and then increases. When the fingerprint dimension *m* = 6, the average positioning error is 0.78 m. Taking this as the inflection point, when the fingerprint dimension is too low, the features pertaining to positioning are also lost; hence, the positioning error is relatively large. When the fingerprint dimension is too high, the noise in the data cannot be effectively removed; this influences the positioning accuracy.

### 4.4. Analysis of Single Fingerprint Positioning Error

**Figure 7:**
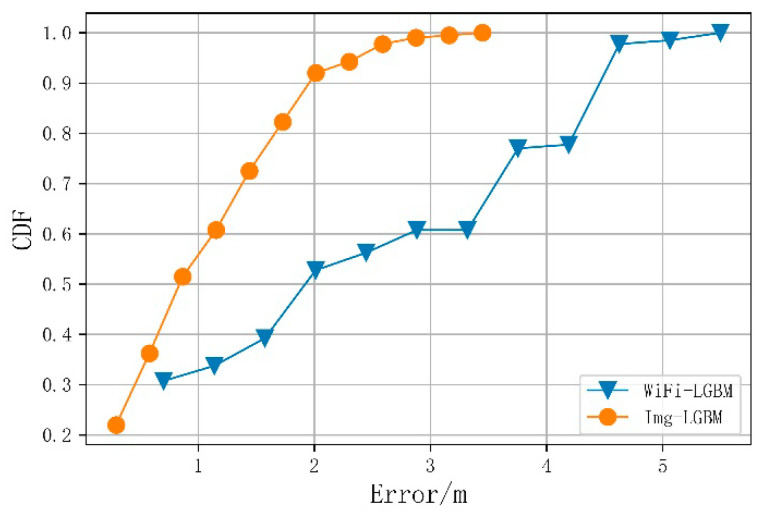
Cumulative probability distribution of positioning error for a single-fingerprint dataset.

For comparison, Table 2 and Figure 7 show the average positioning error of the LightGBM positioning algorithm using the original Wi-Fi fingerprint alone and the image fingerprint alone. The average positioning error of the image fingerprint is 0.97 m, less than the average positioning error of the Wi-Fi fingerprint (2.30 m). Owing to the time variability of Wi-Fi fingerprints and the possible similarities of the teaching building scene, the positioning accuracy of the single-fingerprint dataset was lower than that obtained using a merged one. It can be seen that the complementarity between the merged features is essential for improving the positioning accuracy. When Wi-Fi fingerprints in different locations are similar, these locations are distinguished by image features; conversely, when it is difficult to determine the location for the scene, Wi-Fi fingerprints can provide identification information.

### 4.5. Error Comparison between the Merged-Fingerprint LightGBM Algorithm and Other Positioning Algorithms

This study compared the average positioning error and running time of the merged-fingerprint LightGBM positioning algorithm to verify its effectiveness. The merge-fingerprint LightGBM positioning method was compared with Adaptive Boosting Algorithm (AdaBoost), Decision Tree (DT), Gradient Boosting Decision Tree (GBDT), Random Forest (RF), and Support Vector Regression (SVR). The comparison experiments all use the merged fingerprint dataset with a Wi-Fi fingerprint dimension of 6. 

**Figure 8:**
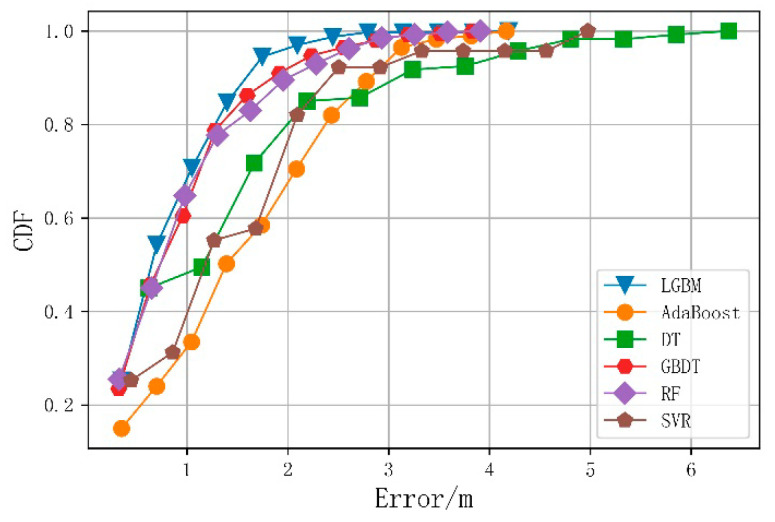
Cumulative probability distribution of the average errors for the six algorithms.

Figure 8 shows the cumulative probability distribution of the positioning errors for the six algorithms after tuning the grid search parameters to reflect the positioning accuracy. It can be seen from Figure 8 that the cumulative probability distribution of the fusion fingerprint LightGBM positioning algorithm in each error range exceeded that of the other algorithms, and a positioning accuracy of 90% was achieved within 1.53 m for all samples. 

Table 3 compares the average positioning errors and running times of the six algorithms when using 50, 80, 90, and 100% of the samples. The final average positioning error of the fusion fingerprint LightGBM algorithm was 0.78 m, more than 15% more accurate than the other five algorithms. The merged fingerprint data proposed in this paper are a type of structured data, and the DT-based model performed better in terms of positioning error. Among the three high-positioning-accuracy algorithms (i.e., LightGBM, GBDT, and RF), the LightGBM positioning model ran fastest, at 16.75 ms. The DT-based ensemble learning model was slower than the DT model, though it fitted the data better. The running time of the proposed algorithm was primarily consumed in the feature extraction stage. The total running time (for extracting WiFi fingerprint KPCA features and image LBP features) was 1.71 s.

### 4.6. The Influence of the Maximum Depth of the Classification Regression Tree on Positioning Accuracy

**Figure 9:**
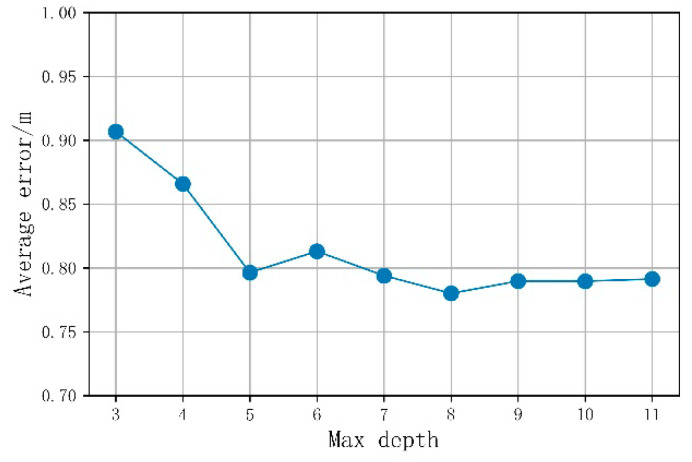
Influence of the maximum depth of the classification regression tree on the average positioning error.

The LightGBM base learner is a classification regression tree. To increase the generalizability of the model and prevent it from overfitting, the maximum depth of the classification regression tree must be limited. As can be seen in Figure 9, the average positioning error first decreases and then increases when the maximum depth of the classification regression tree is increased. When the maximum depth was 8, the curve reached its lowest point, and the average positioning error was 0.78 m. By continuing to increase the maximum depth, the model appeared to over-fit, the generalizability was weakened, and the positioning error increased. The model learning rate was 0.08, the number of classification regression tree was 85, and the maximum number of leaves was 17.

### 4.7. Comparison with Other Algorithms

Table 4 presents a comparison between the proposed algorithm and those reported in other studies. It can be seen from the table that the error of the method proposed in this study is relatively small. The positioning error of the Wi-Vi method (proposed by Huang et al.) after a large number of experiments is very small, and the images taken by this method are limited to exit signs and surrounding scenes. When the number of exit signs is small, Wi-Fi positioning is used. This study considers the effects of illumination and shooting angle on the image. We chose as many angles and lighting conditions as possible when shooting the scene images of the training set. Meanwhile, the images in the test set of their experiment were very different from those in the training set. The time interval between the Wi-Fi fingerprint training set and the test set collection in the experiment was two weeks, and the experimental area was a corridor. The flow of people had a greater impact on the Wi-Fi signal strength, though the proposed algorithm was still effective. Because the experimental environments in different studies differ significantly, only the experimental results given in the literature are listed for comparison.

### 4.8. Threats to Validity and Limitations 

Internal validity: The main threat to internal validity arises from factors that may affect positioning performance. The factors that affect the experimental results include the dimensions of the Wi-Fi fingerprint KPCA feature, the parameter settings of LightGBM, and the quantity of scene-image texture information.

External validity: In this study, all experimental data were collected in static mode, and restrictions were placed on the height and angle of image capture. The density of access points in the environment and the use of different equipment to collect data may affect the results of the experiment.

However, this does not mean that this research can only be applied in static mode. In future work, we will conduct experiments under dynamic conditions. This research requires a large quantity of data to be collected during the model-establishment stage, and the quantity and quality of the data are closely related to the experimental results. Therefore, reducing the fingerprint-collection workload is a key consideration.

## 5. Conclusions

A fusion location fingerprint combining Wi-Fi and image features was proposed, and a LightGBM regression positioning model was established. The algorithm first extracts the KPCA function from Wi-Fi information to eliminate noise. Experiments show that, compared with PCA, KPCA extracts more Wi-Fi fingerprint features and reduces the positioning error by more than 0.5 m. Second, the algorithm extracts LBP rotation-invariant unified pattern features from the scene image and stitches these two features together to form a merged fingerprint. Next, it uses LightGBM to build a regression positioning model and construct a mapping relationship between the merged fingerprint and spatial position coordinates to predict the position coordinates of the points to be measured. We chose to transform the image data into structured data to achieve fusion with Wi-Fi fingerprints in the same dimension; thus, we did not need to calculate large amounts of image data and could reduce the algorithm execution time to within 2 s.

This article compares and analyzes the selection of Wi-Fi positioning functions and positioning algorithms. The experimental results showed that the proposed LightGBM fingerprint fusion positioning algorithm exhibited less error and better environmental adaptability compared with the single-fingerprint positioning one. Compared with the traditional fingerprint positioning algorithm, the average error was reduced by 20%, and the model ran faster than other positioning algorithms. Thus, it represents a simple and effective positioning method. Compared with other similar studies, our model achieves a smaller average positioning error of 0.78 m.

This work is not just suitable for the automatic positioning of pedestrians: it can also be combined with other positioning methods to be implemented in robots. However, the data collection workload is relatively large and experimental scenarios are scarce. Future research directions include the rapid generation of fingerprint databases and the development of adaptive positioning systems.

## Figures and Tables

**Table 1:** Experimental equipment parameters.

Phone Parameters	Values
Phone model	Mi 10 Ultra
CPU	Snapdragon 865 processor
CPU processor	8core 2.84 GHz
GPU	Adreno 650 587 MHz
Wi-Fi (WLAN)	Support Wi-Fi 2.4 G/5 G dual-band, IEEE 802.11 a/b/g/n/ac/ax
OS	MIUI12.0.15

**Table 2:** Comparison of average positioning error for a single-fingerprint dataset.

Localization Algorithm	50% Sample Error/m	80% Sample Error/m	90% Sample Error/m	Average Error/m
Wi-Fi-LGBM	0.82	1.73	2.03	2.30
Image-LGBM	0.36	0.69	0.81	0.97

**Table 3:** Comparison of average positioning errors and running times of the six algorithms.

Localization Algorithm	50% Sample Error/m	80% Sample Error/m	90% Sample Error/m	Average Error/m	Running Time/ms
LGBM	0.32	0.55	0.64	0.78	16.75
SVR	0.54	0.98	1.13	1.38	20.08
DT	0.48	0.87	1.05	1.37	2.64
RF	0.33	0.59	0.72	0.90	35.81
AdaBoost	0.72	1.16	1.32	1.51	17.01
GBDT	0.35	0.61	0.72	0.89	18.55

**Table 4:** Comparison of the proposed algorithm with established algorithms.

Method	Technology	Environment Area	Error in Meters	Error %
Jiao et al. [33]	Wi-Fi, RGB	205 m^2^	0.83	N/A
Huang et al. [16]	Wi-Fi, Vision	12000 m^2^	0.5	5%
Jiao et al. [36]	Vision/wireless/inertial	192 m^2^	1.23	4.4%
Guo et al. [24]	Wi-Fi fingerprint	1460 m^2^	3.4	N/A
Proposed Method	Wi-Fi, Scene image	70 m^2^	0.78	N/A

## Data Availability

The experiment uses an internal data set. The data presented in this study are available on request from the corresponding author.
